# On the Localized Causes of Cholera

**Published:** 1858-10

**Authors:** F. H. Johnson

**Affiliations:** Sunderland.


### TRANSACTIONS

OF THE

### EPIDEMIOLOGICAL SOCIETY OF LONDON.

### Papers and Communications.

### ON THE LOCALISED CAUSES OF CHOLERA.

By F. H. JOHNSON, Esq., Sunderland.

[*Read before the Society on Monday, June 4th,* 1856.]

THE occurrences of the last thirty-six years, seem to indicate the extreme probability that cholera, if it do not become a disease of home growth and indigenous to the soil, is, at least, very likely to shorten the intervals between its visitations, and on each occasion to disseminate more widely its influence upon the bills of mortality.

It seems, therefore, highly desirable, both as a question of pathological interest, and as one upon the settlement of which some groundwork for the establishment of definite sanitary laws might be raised, that there should be more harmonious opinions than those at present adopted, as to the means by which this terrible scourge is propagated among the community. On its first appearance in 1831, when the public mind was excited by its fury and novelty to a point whereat cool observation and deduction must have been extremely difficult, few opinions were free from a certain amount of prejudice or clashing experiences.

The predominating verdict was certainly, at that time, in favour of the contagious nature of the disease; and to this conclusion many were doubtless helped by popular opinion and preconceived notions drawn from the history of other countries. During subsequent visitations some amount of re-action against this feeling had arisen, and after the epidemic of 1849, the sense of the profession, so far as it could be drawn from the evidence and inferences contained in the *Report of the Royal College of Physicians*, was as decidedly against the disease necessarily possessing contagious properties. During the fearful seizure which, in 1853, devastated certain districts in England, professional opinion appeared to have again undergone a revulsion, and a very decided majority of observers drew their conclusions in the presence of numerous cases, which could only be explained upon the grounds of personal transmission; those who doubted, still directing however their precautionary measures in such a way as could only be accounted for by similar opinions partially admitted.

A class intermediate in their views, adopted, *pro tanto*, each opinion; and while they maintained that cholera was neither necessarily developed by contagion, nor spread without it, argued that each case was capable of reproducing the malady, and frequently did so; although during an epidemic seizure of great extent, the spread was by no means necessarily dependent upon personal communication, but went on irrespective of intercourse between the infected districts.

They argued from the analogy of other epidemics, such as scarlatina and smallpox, in which a class of facts identical with those observed in cholera could be adduced; and at the present day, they probably form the great majority.

The object of the following remarks is to show the feasibility of one of these positions, and the chief importance claimed for the facts adduced is, that the observations were made at a time when cholera neither was, nor had been, epidemic in the locality. So that any inferences might be drawn, free from conflicting sources of development.

The town of Sunderland enjoys the dubious honour of having been the first in the United Kingdom in which cholera showed itself, and that in which its virulence was first fully exhibited in this country. It is a large seaport town of about sixty-five thousand inhabitants, situated at some elevation on the sloping limestone banks of a tidal river; it is to a great extent naturally drained, and contains in its more ancient part an area of habitations populated above the average density. Its mercantile communications with the continent, have at all times rendered it liable to the importation of disease, the sanitary regulations bearing upon shipping being almost universally neglected, and the habits of the seamen rendering them prone to receive contagious diseases.

The town has suffered from three attacks of cholera, viz.: in 1831-2, 1848-9, and 1853-4. In the first, 202 died, or one in 200 of the population. In the second, 359, or one in 185. In the third, 28, or one in 2,230.

In all three instances, the first noticed case of the disease was imported by shipping, and on every occasion the subject of the disease had just arrived in port from Hamburgh.

In 1849, after an interregnum of three months health, a fresh outbreak took place subsequently to the introduction of a sick sailor from London, whose mother also perished during her attendance upon him.

In 1853, the cases which were noticed also resulted from intercourse with infested districts, and it is to them chiefly that this paper refers.

The disease known as cholera, will generally be found localized under one or both of two conditions, alternating and varying with the influences which are brought to bear upon its progress. The first, that most generally seen, is its epidemic form; in which the atmospheric or terrestrial influences, which are capable of producing an outbreak, independent of any discoverable communication, are exerted; where, too, there is an antecedent tendency throughout the community towards morbid changes in the secretions closely allied to the general symptoms of the epidemic seizure. Where, in short, the so-called epidemic constitution of the air is in operation, and the disease assumes its most prevalent and fatal form.

The second, and the one whose existence is not so generally admitted, is that accession of cholera which arises from direct communication with infected districts or persons; wherein there is no spontaneous outbreak coexistent in adjoining neighbourhoods, but where distinct groups spring up irrespective of locality, in an apparently consequent chain of causation, and then either merge into a general outbreak from gradual spread, or die out without transgressing the limits of personal communication;—where, in fact, cholera can neither be referred to a sporadic nor epidemic origin, but is distinctly traceable from individual to individual, and increases or decreases in proportion to the extent of personal intercourse.

One object of this paper is to show how far this personal intercourse or communication influences the propagation and spread of the disease.

It has been already remarked, that on every occasion of an outbreak, cholera has been first introduced into Sunderland through the same channel. A case has been imported from a remote infected district, and proved more or less fatal to persons in direct communication with it; but an interval has always existed, during which the instances were more or less solitary before the epidemic or spontaneous eruption took place. The course of the epidemic has been gradual, acquiring a maximum intensity, and then frequently resolving itself, finally, into the scattered groups with which it commenced. Frequently, however, in its decline, the seizures were solitary, unconnected, and when so, always amongst the most severe.

Numerous examples were observed, even during the epidemic, where the transmission of the malady could only be explained on the supposition of its contagious nature; but illustrations most free from objections are to be found where no reasonable explanation intervenes between the association of the disease infecting and the person infected, and the following groups tend towards this proposition. It must be premised, that at the period when these cases occurred, Sunderland was otherwise entirely free from cholera. The neighbouring town of Newcastle-upon-Tyne was undergoing one of the most frightful epidemic seizures on record, and from the hourly intercourse existing, great fears were entertained that Sunderland would, in its turn, partake in the same general influences, which it however never did. Importations only took place, and from them this paper is illustrated; but beyond the record of their consecutive succession, the disease never travelled. For example. The first case noticed in the borough was a sailor landed from Hamburgh, who died, but from whom the disease did not spread.

The next, is a man resident in Sunderland, who visits a sister dying of cholera, in or near Newcastle, and assists about her person. He returns home, within twelve hours sickens, and becomes collapsed, but recovers. A woman residing in the adjoining room, and acting as his nurse, sickens and dies; her husband also perishing, on the following day, from the same cause. The joiner who coffins the body of the latter, is immediately seized and dies; a neighbour occupied about him, also dies. The mother of the second victim on the list perishes within two days; and a female nursing this last is also attacked severely, but recovers, and there the group of disease ends. Now, taking the term contagion in its narrowest sense, viz., an exposure of the healthy structures to contact with those contaminated, the conditions were in these cases fulfilled, inasmuch as in all a mutual handling of the person and inhalation of the breath, or odour of the secretions, took place.

No one beyond the pale of personal communication was attacked, and all who were attacked were placed in direct contact with the bodies of the sick, moving, rubbing and washing them. No other cases occur in the locality, or within ten miles of it, simultaneously with these mentioned.

Again, in an opposite extremity of the town, equally free from epidemic disease, a woman supposed to have been infected in Newcastle was seized; her mother in nursing her, received the seeds of infection and died; a nurse waiting upon her also sickened and perished; two other women assisting in laying out the body also underwent seizures, but recovered. It will be remarked, that the fatality of the seizures in all these cases was somewhat remarkable, and presuming that the disease reproduced is proportionate in severity to the intensity of the cause, there could be little doubt of the saturation to which the victims were subjected.

Taking the question of the transmission of infection in a wider sense, and assuming that contagion may be conveyed through an agency, intermediate between the sick body and the healthy, without actual association, the following facts are worth consideration.

A man sickened after visiting Newcastle and died. I may here mention that the seizure almost invariably became apparent within twelve hours after returning from the infected locality. His clothes were handed over to two women residing on the opposite side of the river, they both took the disease and both died. Another man, a sailor, died at sea, and was cast overboard; his clothes were brought to his friends and handed over to a laundress, who sickened and became collapsed, but recovered. Again, to prove that the mere locality did not influence the operation of the poison. A woman coming from Newcastle sickened at a healthy village; her two daughters residing in another healthy village, distant two miles from this last, visit her, and returning home with her clothes, both sickened and both died. No other cases occur in either of the villages in question. Now, in order to explain this chain of disease; in the first group, where positive contact occurred, without referring the sequence to the transmission of cholera poison from person to person, we must have some reasonable grounds for supposition, that, either a local cause sufficient for the spontaneous development of cholera existed, or that each case was the result of germination, *per se*, from general epidemic causes. That no localized cause, other than the primary source existing in the infected case, existed, seems pretty evident from the fact that the occurrence of the disease was limited to the instances mentioned, amid a dense population equally exposed to such causes; that the malady had a spontaneous or atmospheric origin is equally inexplicable, both from the fact last mentioned, and from the circumstance that no source of infection or epidemic influence was known to be existing, or exerting its presence, within eleven miles of the place in question. The only legitimate inference, surely, is that the man first on the list formed the local cause.

In the second group, where contagion seemed to have been conveyed by the clothing, there is reason to believe that no immediate personal contact took place.

Indeed, in the first case it was improbable, in the second impossible. In the third, there was a probability of both immediate and intermediate contact, but none whatever of sporadic origin.

The first subject brought the disease into a healthy locality, and the daughters returning to one equally uninfected, developed in it a definite form of disease, that disease spreading no farther.

But if cholera had been received from local causes, apart from the infected body, in which of the localities did it exist? Not in that first visited, because it was proved to have been imported there by this single case only; not in the last also, because the disease began and ended with the two cases which conveyed it from the first.

Limited in number as these records appear, they seem to prove all that is proved of other diseases avowedly contagious, and if the proof be admitted in them, where is the line of distinction to be drawn? During the prevalence of an epidemic, doubts as to the source of disease may arise; but, under the circumstances here described, it seems hardly logical to attempt an explanation, except on the grounds of contagious communication. Indeed, of twenty-six authenticated cases, spreading over a period of nine weeks, I cannot find any one independent of contagion received within or out of the town. It would be untrue to allege, that in the full fury of an epidemic outbreak, each case can be traced to its definite source of contagion; but it certainly holds good in this town as regards each incipient epidemic, which has always commenced with isolated cases brought in from infected districts, and extending for some time from these *foci* only. So far as the personal experience of the writer goes, excepting in one solitary instance where the subject was of imbecile intellect, he has never yet had a case of cholera brought under his notice in which he was not able to refer the cause, more or less circumstantially, to pollution from personal communication.

A very important question seems to arise out of this subject, viz., whether these groups of cases contain within themselves the power of converting isolated seizures into an epidemic outbreak. In other words, whether the multiplication of the disease is capable of so saturating the atmosphere with cholera poison, that the pestilence may spread irrespective of personal communication. There is no reason to doubt, although the links of proof soon become too numerous to singularise, that a mere reduplication of foci would be quite sufficient to produce any amount of disease, so long as intercourse was established; but this would hardly account for the spontaneous and isolated seizures which notoriously prevail, and for the travelling of the disease from country to country, without attributing a reproductive power to other causes than those created in the individual. The writer by no means attributes the spread of cholera to contagion only; he merely wishes to demonstrate that contagion is a frequent—perhaps the most common—cause of its spread. One apparent proof that the morbid germs may be reproduced in the system affected, occurred in a case wherein the man labouring under the disease was completely deprived of the clothes in which he underwent the seizure, and yet those in which he passed through the course of the attack conveyed the contagion to other individuals.

Now, taking the whole of the facts here stated, and transferring to them the heading of "Transmission of Scarlatina or Smallpox," would the mode of spread have been questioned? There is, at least, no other means by which cholera can be propagated, the explanation of which so nearly approaches positive demonstration, as that which points out its transmission from individual to individual, by the evolution and reception of a communicable *materies morbi*. There is, too, something extremely significant in the fact, that along the east coast, equally exposed at every point to atmospheric imports, the point of landing, development, and spread should be that at which personal communication was most probable and best known to exist.

The practical question which naturally arises out of all this is, "If cholera can thus be spread by personal intercourse, what are the best means to adopt for its suppression, and from what quarter is the danger to be most apprehended?" If cholera multiply itself in proportion to the number of persons exposed to its presence, it is clearly the duty of public bodies to direct their attention first to its limitation. The great avenue by which danger enters into every seaport town is its river, from which are landed the cases destined to form each its *nidus* of contagion. During the epidemic of 1853, when the cases here detailed were noticed, the writer had charge of the river Wear and its shipping, amounting, on one occasion, to seven hundred vessels and 10,000 men. He established an examination of each ship as it arrived; had all suspicious vessels cleansed and purified; personally inspected all cases of illness reported; removed the sick into healthy quarters; formed depôts of medicines at sailors lodging-houses, grog-shops, and other places of resort, at which any man complaining might have a dose administered with the spirit for which he was sure to apply; and by dint of enforcing the almost obsolete regulations of the Board of Trade, as regards seamen's accommodation, contrived to get unwholesome feeding and sleeping places rid of lumber and dirt. Out of two hundred and forty-three cases of diarrhœa, only one of well-marked cholera took place, and has recovered without spreading the mischief. Of purifiers there is probably nothing so good on board ship as free fumigation with sulphur, and a thorough ablution with water. The proceeding most valuable in its results, although the most difficult to carry out, is the arrest of communication between the sick and healthy. To collect together in one building a number of patients labouring under any contagious disease, seems but to concentrate and intensify the contamination,—it might be to convert a casual visitation into a certain epidemic. The system found most practicable and most satisfactory in its results at Sunderland, has been to limit as far as possible the number of attendants, allowing the patient to remain in the locality where he was seized, provided no sanitary reasons opposed it; to destroy as soon as possible all bedding, clothing, and contaminated linen; to freely purify and deodorise the locality, keeping, at the same time, a sedulous watch over those about, and submitting any incipient symptoms to instant treatment. Scavenging was proceeded with cautiously, inasmuch as the disturbance of accumulated filth might produce the mischief most feared; all foul heaps and sewers were first thoroughly covered up with lime, and allowed to remain so for some days before removal, which was done gradually. Running water and surface-drainage ought always to be regarded with suspicion. It is by no means an extravagant hypothesis, that the elements of contagion contained in the discharges of the sick, and conveyed away by open drains or disgorged from sewers, may be a frequent channel through which cholera is circulated among the ramifications of a town.

Quarantine regulations being quite impracticable in British commerce, need not be here noticed; a means quite as efficacious, by being more under personal control, is afforded in the careful management and superintendence on shore of the cases as they occur. Arrangements for providing the poor with speedy attention are amongst the most valuable of all provisions. It is within the experience of every medical man that cholera, when promptly treated, is usually a tractable disease, but that neglect too often converts trivial cases into a malignant form. Moreover, there is a singular reluctance in those seized with the first symptoms of the disease to seek advice; and when they do so, unless the relief be immediate, the disease becomes unmanageable with singular rapidity. Local depôts, therefore, both by giving ready assistance, and by quieting the apprehensions of the population, afford both moral and therapeutic support. The powers possessed by Local Boards to carry out fully and completely such arrangements as these mentioned, are frequently inadequate, and sometimes clash. What the law ought to provide, and what is now to some extent afforded by the Public Health and Nuisances Removal Acts, is authority to the municipal and parochial boards to form one sanitary board, giving them absolute and immediate powers to remove all nuisances, to take charge of all districts pronounced infected, to appoint surveillance over individual cases, and to provide gratuitous advice and medicines for all applicants.

In 1853, the town of Newcastle lost, by cholera, one in forty of its population. At Sunderland, in hourly communication with it, the disease never assumed a form strictly epidemic, and the mortality was one in 2,230 of the population. What was done to aid in procuring this exemption amounted to the precautions already alluded to. The town council and the board of guardians formed a united and unanimous Board of Health, not in name only, but one divided into sections; and each section undertaking the charge of surveying and superintending a subdivided locality.

They employed scavengers, whitewashers, and inspectors; instituted medical surveillance over infected districts; and where the law was deficient in its powers, they took the place of the law.

The whole corps was headed by Dr. Joseph Brown, one of the oldest and most experienced observers of the disease, and all the arrangements were carried out by the townspeople themselves.

The whole extra expense of these preceedings amounted to £524 : 6s, or about 1¼d. in the pound on the rateable value of the borough. The result appeared to be that each focus of disease only exerted a limited influence, that its progress was arrested at every point, and under circumstances apparently similar to those in which it had on previous occasions advanced.

It is not easy to prove how far imperfect drainage or sewage facilitates the transmission or spread of cholera, although abundant evidence exists of its aiding in the production of sporadic cases.

As regards the instances in question, in all the drainage and sewage were extremely deficient.

In the first group all, save one, lived in dwellings quite destitute of drainage, privies, or ashpits; and it was by no means uncommon to find the contaminated utensils placed under the sick bed, or stationed on the landing.

The exception was the last person in the series, who was removed after the seizure to an open and clean neighbourhood, and it was with her that the chain of disease dropped. The slope of a hill, upon the alluvial soil covering the limestone, was in all these cases the physical characteristic of the locality; but in no epidemic did the development of the cholera appear to prefer the foot of the descent to the localities from that to half-way up the rise. The vicinity of the river did not remarkably affect the local mortality.

The wide streets, higher-class houses, and the districts situated upon the sand and limestone, were most exempt; in fact, seemed to be surrounded by an invisible defence beyond which cholera never, in one single instance, on any occasion passed, among a population of five or six thousand people.

The density of the population appears to be a most important local cause of spread in this as in every other epidemic disease. The sub-epidemic herein especially alluded to, is not calculated to afford much information on the subject, but the following figures drawn from previous visitations are highly instructive. The town of Sunderland is divided into the parishes of Bishop Wearmouth, Sunderland, and Monk Wearmouth, the river dividing the latter from the two first.

Bishop Wearmouth contained at the last census, 28,846; Sunderland, 17,020; Monk Wearmouth, 12,362. But in 1831, the numbers were about as follows:—Bishop Wearmouth, 14,825; Sunderland, 17,060; Monk Wearmouth, 5,310. In Bishop Wearmouth, there are about 1,900 acres, inhabited space; in Sunderland, 133 acres; in Monk Wearmouth, 1,566 acres. In Bishop Wearmouth, therefore, each individual would enjoy 623 square yards of space; in Monk Wearmouth, each 1,427 yards, and in Sunderland, each 38 square yards.

Now, during the epidemic cholera of 1831, the deaths were, in Bishop Wearmouth, one in 706; in Monk Wearmouth, one in 354; in Sunderland, one in 109.*

The discrepancy between the relative mortality of Bishop and Monk Wearmouth must be accounted for, to some extent by the fact, that the latter, at that time, consisted chiefly of a badly constructed neighbourhood, inhabited by a poor population.

In 1849, the figures as regards cholera, showed thus:—Bishop Wearmouth, with a population of one individual to each 260 square yards, lost one person in 448. Monk Wearmouth, with one to 505 square yards, lost one in 294.

Sunderland, with one to 38 square yards, lost one in 73.

In other words, a density of population represented by seven, had six times the mortality of that represented by one.

Or, taking another view of the population. Each house in the parish of Sunderland contained about ten persons; each in Bishop Wearmouth, about five. In 1831, Sunderland lost 202; Bishop Wearmouth, 22. In 1849, Sunderland lost 231; Bishop Wearmouth, 77.

In an epidemic of measles in 1824, Sunderland lost 222; Bishop Wearmouth, 123. These numbers bear a curiously-close resemblance to the relative density of the population, in this, an avowedly contagious disease. From this, it would appear that mere proximity of man to man, is favourable to the spread of cholera; a fact, certainly consistent with the supposition of its contagious nature.

---

* These figures are chiefly taken from the article on Cholera, in the "Cyclopædia of Medicine", by Dr. J. Brown.

Moreover, there is certainly no other disease in which the same facilities are offered for the transmission of such contagious properties as may be contained in the secretions, from their enormons amount and the rapidity with which they are thrown off. The writer has not observed any particular class of occupation more or less liable than the rest to cholera, with the exception that among those working in a heated atmosphere, such as pitmen, glass-makers, and the like, the disease appeared to spread more rapidly, and be more fatal in its results. Sailors, pilots, ballast-heavers, coal-trimmers, and those whose labour took them most frequently among infected vessels, were always the first to be attacked in the epidemic.

The proportions borne by the sexes were as follows:—in 1849, of 343 fatal cases, there were 156 males, and 187 females. Of the males 74, and of the females 81, were between 20 and 50 years of age.

The preponderance of mortality in the female sex, the writer is disposed to attribute to the readiness with which the duties of nursing the sick are fulfilled by them, and the consequent exposure to contagion they incur. It may be also mentioned, that the town of Sunderland has an almost unlimited supply of the purest water, obtained from a loose quicksand overlaying the coal measures and beneath the upper strata of magnesian limestone, at a distance of nearly two miles from the town. The water is highly charged with free carbonic acid gas, and the supply is upwards of two million gallons daily. It was quite impossible that the drinking supply of the town could have in any way become contaminated, by filtration; almost every house being supplied with a separate tap of its own.

The writer has endeavoured to enumerate, so far as they have occurred to him, the chief local causes which influence the spread of cholera, or are best calculated to arrest its progress. His experience is a very minute fragment; but it is from such, that the great world of science has been created.

